# Gut microbiota alterations associated with anxiety and depression in inflammatory bowel disease: a systematic review of prospective cohorts and randomized trials

**DOI:** 10.3389/fmicb.2026.1853009

**Published:** 2026-06-02

**Authors:** Tao Zhang, Lin Wang, Zhetan Ren, Ru Man, Xiaozhen Cheng, Ling Wang, Jing Wang, Xiumei Bu, Yongduo Yu

**Affiliations:** 1 Liaoning University of Traditional Chinese Medicine, Shenyang, China; 2 Department of General Surgery, Beijing Shijitan Hospital, Capital Medical University, Beijing, China; 3 Shenzhen Traditional Chinese Medicine Hospital, Shenzhen, China; 4 Second Affiliated Hospital, Liaoning University of Traditional Chinese Medicine, Shenyang, China

**Keywords:** anxiety, gut microbiota, gut–brain axis, inflammatory bowel disease, systematic review

## Abstract

**Background:**

Anxiety and depression are highly prevalent among patients with inflammatory bowel disease (IBD) and are closely associated with reduced quality of life and poor treatment adherence. The gut–brain axis has been proposed as a key mechanistic framework, with the gut microbiota playing a central role. However, findings from clinical studies remain inconsistent, and a comprehensive synthesis of the evidence is lacking.

**Objective:**

This systematic review aimed to integrate evidence from randomized controlled trials (RCTs) and prospective cohort studies to evaluate the associations between gut microbiota characteristics, microbiota-targeted interventions, and symptoms of anxiety and depression in patients with IBD.

**Methods:**

A systematic search was conducted in PubMed, Embase, the Cochrane Library, Web of Science, EBSCOhost, and Scopus. The search covered records from database inception to October 17, 2025. The review was conducted in accordance with the Preferred Reporting Items for Systematic Reviews and Meta-Analyses (PRISMA) guidelines. A descriptive synthesis approach was used to summarize the findings. Study quality was assessed using the Critical Appraisal Skills Programme (CASP) checklist.

**Results:**

Ten studies were included, comprising six prospective cohort studies and four RCTs. These studies included a total of 1,040 patients with IBD. Cohort studies consistently showed that anxiety and depressive symptoms were associated with reduced microbial α-diversity, enrichment of pro-inflammatory taxa, and depletion of short-chain fatty acid (SCFA)-producing bacteria. These associations persisted after adjustment for inflammatory markers. They were also observed when analyses were restricted to patients in remission. This suggests that the associations may not be fully explained by disease activity. Evidence from RCTs showed that microbiota-targeted interventions, including probiotics and fecal microbiota transplantation, modulated microbial composition and reduced anxiety and depression scores. Studies combining psychological interventions with microbiota profiling also suggested potential effects on gut microbial composition. However, the findings remain heterogeneous.

**Conclusion:**

Current evidence supports a close and potentially bidirectional association between gut microbiota dysbiosis and anxiety and depression in patients with IBD. Both microbiota-targeted interventions and psychological therapies may represent promising strategies for managing psychological comorbidities in IBD. Future large-scale, standardized longitudinal studies and randomized controlled trials are warranted to clarify directionality, test causal hypotheses, and develop personalized intervention approaches.

**Systematic review registration:**

https://www.crd.york.ac.uk/PROSPERO/, identifier CRD420251166542.

## 1 Introduction

Inflammatory bowel disease (IBD), including ulcerative colitis (UC) and Crohn’s disease (CD), is a group of diseases characterized by recurrent episodes of chronic intestinal inflammation (Kaplan, 2025). Globally, the prevalence of IBD continues to rise, particularly in industrialized countries and emerging regions, with a trend towards younger onset (Hracs et al., 2025). Although drug therapy and the use of biologics have significantly improved the clinical outcomes of IBD, patients commonly experience symptoms such as anxiety and depression, far exceeding the general population (Mudra Rakshasa-Loots et al., 2025; Riggott et al., 2025). These psychological symptoms not only affect patients’ quality of life and treatment adherence but may also exacerbate intestinal inflammation through the neuro-microbe axis, creating a vicious cycle (Chen et al., 2025). In recent years, research on the gut-brain axis has revealed the important role of gut microbiota in regulating host mood and neurological function. Numerous basic and clinical studies have shown that dysbiosis may contribute to anxiety and depression-like behaviors by affecting neurotransmitter metabolism, inflammatory responses, and hypothalamic-pituitary-adrenal axis activity (Tiwari and Paramanik, 2025). Altered gut microbiota composition has also been widely reported in patients with IBD. These alterations include reduced microbial diversity and enrichment of specific bacterial taxa. They are closely associated with disease activity and the severity of psychological symptoms (Cortés et al., 2025; Iliev et al., 2025).

Currently, there is an increasing number of clinical studies on the relationship between gut microbiota and IBD-related anxiety and depression, including randomized controlled trials (RCTs) and prospective cohort studies. Interventions include probiotics, prebiotics, fecal microbiota transplantation (FMT), and dietary regulation. However, due to limitations in study design, microbiota detection methods, psychological assessment tools, and sample heterogeneity, different studies have yielded conflicting conclusions, and a systematic quantitative analysis is still lacking (Ananthakrishnan et al., 2025; Hu et al., 2025).

This systematic review aimed to synthesize evidence from RCTs and prospective cohort studies. We assessed the associations between microbiota-targeted interventions, gut microbiota characteristics, and symptoms of anxiety and depression in patients with IBD. To our knowledge, this is the first systematic review to integrate evidence from both RCTs and prospective cohort studies on this topic. By integrating clinical and microbiological data, this review may help clarify the role of the gut–brain axis in psychological comorbidities of IBD. It may also provide evidence for individualized treatment strategies, psychological management, and future research.

## 2 Methods

This systematic review was conducted in accordance with the Preferred Reporting Items (PRISMA) statement for systematic reviews and meta-analyses (Page et al., 2021). This study was registered on the PROSPERO International Systematic Reviews Registry (https://www.crd.york.ac.uk/PROSPERO) (CRD420251166542).

### 2.1 Search strategy

A comprehensive literature search was performed across six electronic databases—PubMed, Embase, the Cochrane Library, Web of Science, EBSCOhost, and Scopus—from database inception to October 17, 2025. Only studies published in English were considered. The primary search terms included “Inflammatory Bowel Diseases,” “Crohn Disease,” “Ulcerative Colitis,” “Gastrointestinal Microbiome,” “Microbiota,” “Depression,” “Anxiety,” “Mood Disorders,” and “Stress, Psychological.” These terms were combined using the Boolean operator “AND,” while related terms within each concept (IBD, gut microbiota, and anxiety/depression) were combined using “OR.” The full search strategy is provided in Supplementary material.

### 2.2 Study selection and inclusion criteria

Studies are considered eligible if they meet the following criteria: (1) Study design: human study, cohort study, and randomized controlled trial; (2) Population: adults with IBD; (3) Exposure factors: to investigate the effects of anxiety or depression on the gut microbiota of IBD, or conversely, the effects of gut microbiota on anxiety or depressive symptoms; (4) Outcome measures include at least one of the following: incidence of anxiety and depression, progression, and gut-related biomarkers of anxiety and depression progression; (5) Publication type: peer-reviewed full-text article.

Studies were excluded if they fell under the following categories: (1) duplicate publications; (2) full texts were unavailable or contained high bias (e.g., poorly designed trials, lack of participant data, etc.); (3) reviews, observational studies, case reports, letters to editors, or conference abstracts.

**FIGURE 1:**
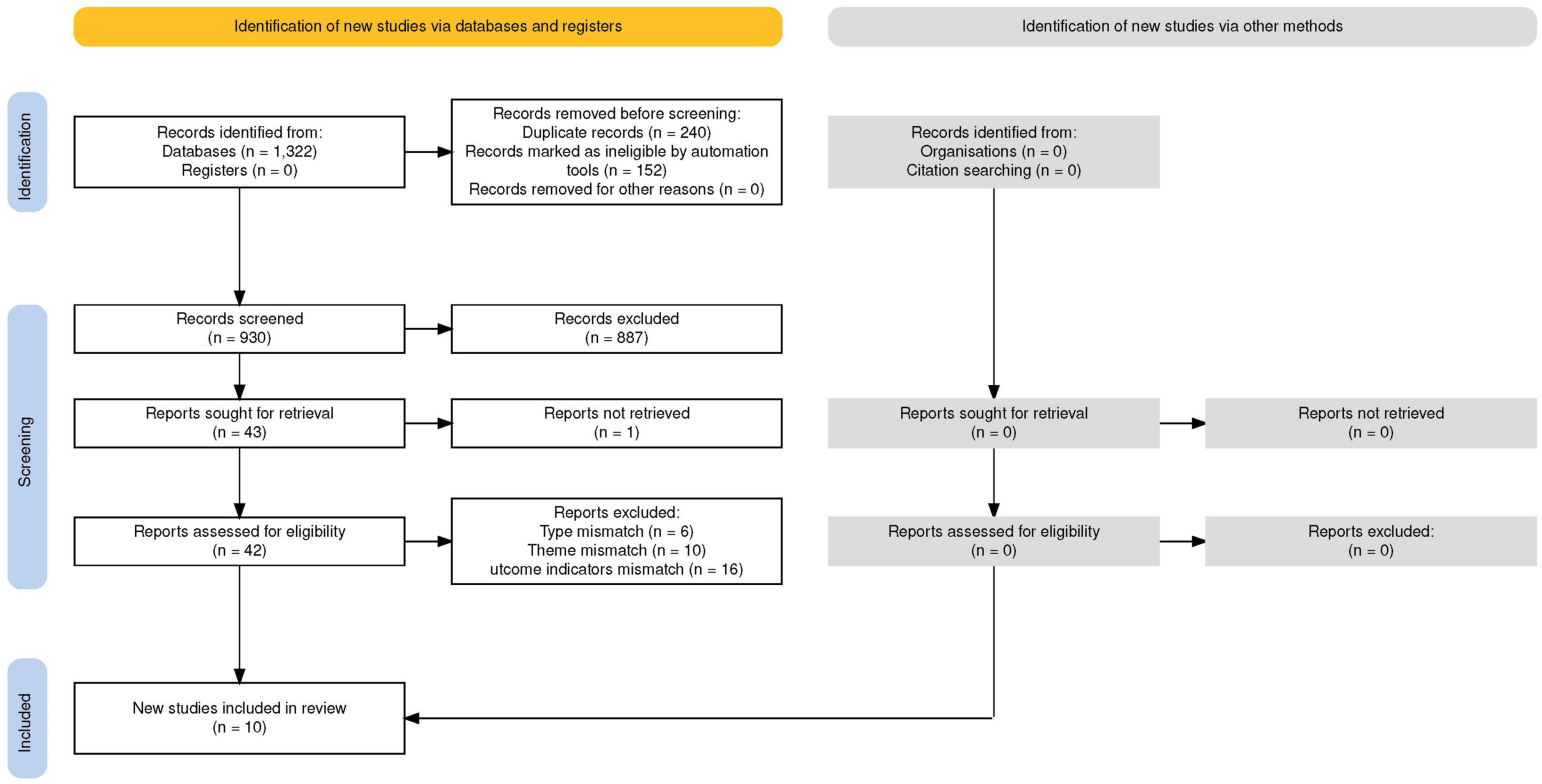
Flowchart of literature screening.

Table 1 shows the specific screening criteria. Duplicate publications were removed after a comprehensive search of multiple databases. Next, titles and abstracts were screened, followed by full text screening based on inclusion/exclusion criteria. The screening and selection process was conducted independently by two reviewers (Tao Zhang and Lin Wang). Any discrepancies were resolved by consensus. A total of 10 studies were ultimately included in the full text review (Figure 1).

**TABLE 1:** PICOS criteria for inclusion of studies.

Principle	Content
Population	Adults diagnosed with inflammatory bowel disease
Intervention	The influence of anxiety or depression on the gut microbiota of IBD, or conversely, the influence of the gut microbiota on anxiety or depression symptoms
Comparator	Control group/No intervention
Outcomes	The incidence, progression, and intestinal-related biomarkers of anxiety and depression progression
Study design	Human studies, cohort studies and randomized controlled trials

### 2.3 Data extraction

Two reviewers (Xiaozhen Cheng and Ling Wang) independently extracted data from 10 studies using Excel, including the following: (1) Study information: first author, publication year, country, study design, sample size; (2) Study subjects: participant characteristics, including age and IBD type; (3) Exposure factors: gut microbiota, gut microbiota assessment methods (and index scoring criteria/biomarkers); 4) Outcomes: baseline incidence and progression of anxiety and depression.

### 2.4 Quality assessment

Two reviewers, Zhetan Ren and Ru Man, independently assessed the risk of bias using the Critical Appraisal Skills Programme (CASP) checklist. The CASP checklist was applied to both cohort studies and randomized controlled trials. For cohort studies, the assessment focused on the validity of the results, the nature of the results, and the local applicability of the findings. Local applicability referred to whether the results could be applied to the relevant clinical setting or population. For randomized controlled trials, the assessment focused on study design, methodological quality, results, and local applicability. Each checklist item was answered as “yes,” “no,” or “not sure.” A high number of “not sure” responses indicated that the reliability of the study findings should be interpreted with caution. The applicability score was calculated as the number of “yes” responses divided by the total number of checklist questions for each study. A higher score indicated better study quality. Disagreements between reviewers were resolved through discussion.

### 2.5 Data synthesis

In this review, we initially planned to perform a quantitative meta-analysis. This analysis would have been conducted if at least two studies reported comparable exposures or interventions, microbiota-related outcomes, psychological outcomes, and extractable effect estimates. However, after full-text assessment and data extraction, quantitative synthesis was considered infeasible. This was due to substantial clinical, methodological, and statistical heterogeneity across the included studies.

First, the included studies differed markedly in study design, IBD subtype, disease activity status, exposure or intervention type, comparator definition, and follow-up duration. The interventional studies also used heterogeneous strategies. These included probiotics, fecal microbiota transplantation, cognitive behavioral interventions, mindfulness-based interventions, and usual care comparisons. Second, microbiota-related outcomes were assessed and reported using different approaches. These included alpha diversity, beta diversity, specific bacterial taxa, microbiome health indices, microbial dysbiosis indices, predicted or measured functional pathways, bile acid profiles, metabolomic signatures, and proteomic features.

Third, psychological outcomes were measured using different instruments. These included HADS, SAS, SDS, PHQ-9, GAD-7, DASS, PSS, and quality-of-life-related scales. These differences prevented direct harmonization of outcome metrics. In addition, statistical reporting varied substantially across studies. Many studies reported the direction of association, taxon-specific differences, correlations, or multi-omics signatures. However, they did not consistently report standardized effect sizes, standard errors, confidence intervals, or sufficient raw data for recalculation. As a result, no single exposure–outcome or intervention–outcome comparison included enough statistically comparable studies to generate a reliable pooled estimate.

Therefore, pooled effect sizes were not calculated. I^2^ statistics, subgroup analyses, sensitivity analyses, and publication bias assessment were also not performed, because these analyses were not appropriate for the available evidence.

Accordingly, this study used a structured descriptive synthesis approach. We summarized the findings according to study design, microbiota domain, psychological outcome, intervention type, statistical reporting, and evidence direction. The consistency and limitations of the available evidence were presented in both tabular and textual formats.

## 3 Results

### 3.1 Overview of included studies

A total of 10 studies were included in this systematic review. These included six prospective cohort studies (Chen et al., 2021; Feng et al., 2022; Humbel et al., 2020; Scaldaferri et al., 2023; Thomann et al., 2022; Yuan et al., 2021) and four randomized controlled trials (RCTs) (Ewais et al., 2021; K et al., 2024; Liu et al., 2025; Shang et al., 2023). Together, these studies involved 1,040 patients with IBD, including UC and CD. The cohort studies mainly examined the associations between gut microbiota characteristics and symptoms of anxiety and/or depression. The RCTs evaluated the effects of microbiota-related interventions on microbial composition and psychological outcomes. The detailed characteristics of the cohort studies and RCTs are summarized in Tables 2, 3, respectively. To facilitate comparison of the core evidence, we also summarized the main microbiota-related findings, psychological outcomes, statistical reporting, and evidence direction in a standardized evidence summary table (Table 4).

**TABLE 2:** Summary of a prospective cohort study on the different levels of anxiety and depression and the characteristics of intestinal flora in IBD patients.

Study (year)	Country	IBD type	Sample size	Disease activity	Follow-up duration	Psychological assessment tool(s)	Main findings: microbiota and psychological symptoms
Scaldaferri et al. (2023)	Italy	Ulcerative colitis	39 UC patients; 37 healthy controls	Mixed (active and remission)	Ongoing follow-up	MMPI-2, STAI Y1/Y2, HADS, PGWBI, GSE, CD-RISC, TAS-20, GSCS	1. Key microbiota markers: Elevated levels of Enterobacteriaceae, Streptococcus, and Veillonella, along with decreased levels of Klebsiella and Clostridiaceae, are associated with anxiety/depression. 2. In UC, there are increased levels of Actinobacteria, Proteobacteria, and TM7; and decreased levels of Verrucomicrobia, Euryarchaeota, and Tenericutes.
Humbel et al. (2020)	Switzerland	Crohn’s Disease and Ulcerative Colitis	171 IBD patients in remission	Remission only	Not reported	HADS, PSQ, SF-36, IBDQ	1. A higher level of perceived stress is associated with lower alpha diversity. 2. Anxiety and depression are significantly associated with beta diversity. 3. A decrease in Clostridia, Bacilli, Bacteroidia, and Beta/Gamma - proteobacteria is associated with psychological distress. 4. A decrease in Lachnospiraceae, Ruminococcaceae, and Veillonellaceae is correlated with anxiety/depression. 5. An increase in Bifidobacterium (CD) and Desulfovibrio (UC) is correlated with depression. 6. An increase in Sutterella, RF32, and Lactococcus is correlated with quality of life (QoL) in Crohn’s disease (CD).
Feng et al. (2022)	China	Crohn’s disease	39 CD patients; 14 healthy controls	Mixed(active and remission)	6 months	SAS, SDS	1. Decreased secondary bile acids in feces and serum; increased 7—DHCA in serum of patients with psychological disorders. 2. Fecal HDCA and 12—DHCA are inversely correlated with SDS; serum 7—DHCA is positively correlated with SDS. 3. Fecal TDCA, TLCA, TβMCA are positively correlated with SAS. 4. Reduced alpha diversity in patients with psychological disorders. 5. Elevated Ruminococcus gnavus, increased Enterobacteriaceae, and elevated Lachnospiraceae in CD patients with psychological disorders.
Yuan et al. (2021)	China	UC	240	Active disease	Not reported	PHQ-9, GAD-7	1. UC with depression/anxiety showed lower microbial richness/diversity; 2. increased Lactobacillales, Sellimonas, Streptococcus, Enterococcus; decreased Prevotella_9, Lachnospira; elevated glycochenodeoxycholate; decreased 2’-deoxy-D-ribose and L-pipecolic acid; reduced immunoglobulin proteins. 3. Prophylactic 2’-deoxy-D-ribose and L-pipecolic acid reduced depressive-like behaviors in colitis mice.
Thomann et al. (2022)	Germany	Crohn’s disease and ulcerative colitis	62	Active disease	Not reported	HADS-D, WEIMuS	1. Depression associated with lower abundance of Odoribacter, Alistipes, Anaerotruncus and reduced pectin/glycosaminoglycan metabolism. 2. Fatigue associated with lower Intestinimonas, Eubacterium, Anaerotruncus, Clostridiales g.i.s. and altered amino acid/carbohydrate metabolism. 3. No association with alpha diversity or inflammatory markers.
Chen et al. (2021)	China	UC	93	Active disease	Not reported	SDS	1. UC with depression had lowest microbial abundance and diversity; decreased Firmicutes, Clostridia, Clostridiales; increased Proteobacteria, Gammaproteobacteria, Bacilli, Enterobacteriales, Lactobacillales; reduced Lachnospiraceae, Prevotellaceae, Roseburia; increased Ruminococcaceae, Enterobacteriaceae, Escherichia-Shigella, Enterococcus.

IBD, Inflammatory Bowel Disease; UC, Ulcerative Colitis; CD, Crohn’s Disease; HC, Healthy Control; MMPI-2, Minnesota Multiphasic Personality Inventory-2; STAI, State-Trait Anxiety Inventory; HADS, Hospital Anxiety and Depression Scale; HADS-A, Hospital Anxiety and Depression Scale-Anxiety subscale; HADS-D, Hospital Anxiety and Depression Scale-Depression subscale; PGWBI, Psychological General Well-Being Index; GSE, General Self-Efficacy Scale; CD-RISC, Connor-Davidson Resilience Scale; TAS-20, Toronto Alexithymia Scale-20; GSCS, Gastrointestinal Symptoms Rating Scale; PSQ, Perceived Stress Questionnaire; SF-36, 36-Item Short Form Health Survey; IBDQ, Inflammatory Bowel Disease Questionnaire; SAS, Self-Rated Anxiety Scale; SDS, Self-Rated Depression Scale; CDAI, Crohn’s Disease Activity Index; MTWAI, Modified Truelove and Witt’s Activity Index; LCA, Lithocholic Acid; DCA, Deoxycholic Acid; HDCA, Hyodeoxycholic Acid; 7-DHCA, 7-Dehydrocholic Acid; 12-DHCA, 12-Dehydrocholic Acid; TDCA, Taurodeoxycholic Acid; TLCA, Taurolithocholic Acid; TβMCA, Tauro-beta-muricholic Acid; GCA, Glycocholic Acid; α-diversity, Alpha diversity; β-diversity, Beta diversity; SCFA, Short-Chain Fatty Acid; LPS, Lipopolysaccharide; TM7, Saccharibacteria; HC, Healthy control; MDD, Major depressive disorder; PHQ-9, Patient Health Questionnaire-9; GAD-7, Generalized Anxiety Disorder Scale-7; WEIMuS, Würzburg Fatigue Inventory for Multiple Sclerosis; SDS, Self-Rating Depression Scale; SCFA, Short-chain fatty acids; fCal, Fecal calprotectin; CRP, C-reactive protein; g.i.s., genus incertae sedis.

**TABLE 3:** The levels of anxiety and depression and the characteristics of intestinal flora in IBD patients in randomized controlled trials.

Study (year)	Country	IBD type	Sample size (E/C)	Intervention	Control	Disease activity at baseline	Microbiota outcome	Psychological outcome	Main results	Follow-up post-intervention
Liu et al. (2025)	China	UC	26/26	Probiotic LAB, 2 capsules × 3 times/day, 8 weeks	Mesalazine alone	Mild to moderate UC	Increased Firmicutes abundance; elevated Firmicutes/Bacteroidota ratio; higher GMHI; lower MDI; more SCFA-producing bacteria; fewer pathobionts	Anxiety: 65.4% improvement; Depression: 53.8% improvement	1. Probiotic LAB improved anxiety and reduced MES; 2. microbiota changes correlated with improved disease activity and psychological status; 3. emotional disturbance improvement facilitated probiotic’s efficacy on disease activity.	8 weeks
K et al. (2024)	Israel	CD	24/25	Cognitive Behavioral and Mindfulness with Daily Exercise, 3 months	Wait-list	Mild to moderate CD	Increased Deferribacteres; decreased Streptococcaceae; decreased Coriobacteriaceae; decreased Lachnospiraceae; increased Mucispirillum; no significant change in alpha/beta diversity; trend toward improved beta diversity vs. worsening in controls	Decreased Depression; decreased Stress; increased Quality of life; decreased PSS4; decreased GSI	COBMINDEX associated with microbial taxa changes correlating with reduced inflammation and psychological distress; baseline Firmicutes correlated with psychological markers	3 months
Ewais et al. (2021)	Australia	IBD (CD/UC mixed)	33/31	Mindfulness-Based Cognitive Therapy, 8 weekly sessions, 2 hours each	Treatment as usual	Remission or mild disease	Shannon diversity index: non-significant increase in both groups; limited samples due to high attrition	Decreased Depression; decreased Stress; increased Active coping; increased Mindfulness; increased Positive reframing; increased Planning	MBCT feasible and beneficial for AYAs with IBD; improved depression, stress, mindfulness and adaptive coping; high attendance but high attrition for biological samples; microbiome changes non-significant	8 weeks and 20 weeks
Shang et al. (2023)	China	UC	68/68 (males); 68/68 (females)	FMT, 9 weeks	Placebo	Active UC	1. increased Species abundance and diversity; 2. male: decreased Clostridiales, Desulfovibrionaceae; increased Prevotella, Lactobacillus, Bifidobacterium; 3. female: decreased Escherichia-Shigella, Desulfovibrionaceae, Staphylococcaceae; increased Porphyromonadaceae, Prevotella, Lactobacillus, Bifidobacterium	Anxiety and depression decreased; The improvement was greater in women	FMT improved clinical symptoms and reduced hsCRP in both sexes; microbiota changes sex-specific	9 weeks

ACT, Acceptance and Commitment Therapy; AYA, Adolescents and Young Adults; BDNF, Brain-Derived Neurotrophic Factor; CI, Confidence Interval; CISS, Chronic Illness-related Shame Scale; CompACT, Comprehensive assessment of Acceptance and Commitment Therapy processes; COBMINDEX, Cognitive Behavioral and Mindfulness with Daily Exercise; CRP, C-Reactive Protein; DASS, Depression, Anxiety and Stress Scale; E/C, Experimental/Control; FCP, Fecal Calprotectin; FFMQ, Five Facets of Mindfulness Questionnaire; FMT, Fecal Microbiota Transplantation; GHQ, General Health Questionnaire; GMHI, Gut Microbiome Health Index; GSI, Global Severity Index; HADS, Hospital Anxiety and Depression Scale; HBI, Harvey-Bradshaw Index; HPA, Hypothalamic-Pituitary-Adrenal; hsCRP, high-sensitivity C-Reactive Protein; IBD, Inflammatory Bowel Disease; IBDQ, Inflammatory Bowel Disease Questionnaire; IL-6, Interleukin-6; ITT, Intention-To-Treat; LAB, Lactic Acid Bacteria; MBCT, Mindfulness-Based Cognitive Therapy; MDI, Microbial Dysbiosis Index; mITT, modified Intention-To-Treat; MMS, Modified Mayo Score; NF-κB, Nuclear Factor kappa B; NLR, Neutrophil-to-Lymphocyte Ratio; ns, not significant; OTU, Operational Taxonomic Unit; PCA, Principal Component Analysis; PCoA, Principal Coordinate Analysis; PLR, Platelet-to-Lymphocyte Ratio; PP, Per Protocol; PSS4, Perceived Stress Scale-4; RR, Risk Ratio; SCFA, Short-Chain Fatty Acid; SCCAI, Simple Clinical Colitis Activity Index; SCS, Self-Compassion Scale; SDS, Self-Rating Depression Scale; SIBDQ, Short Inflammatory Bowel Disease Questionnaire; TAU, Treatment As Usual; TNF-α, Tumor Necrosis Factor-alpha; UCEIS, Ulcerative Colitis Endoscopic Index of Severity.

**TABLE 4:** Standardized evidence summary of microbiota–psychological associations and intervention effects in IBD.

Study	Study design	Comparison/ Intervention	Microbiota domain	Key microbiota finding	Psychological outcome	Statistical reporting	Evidence direction
Scaldaferri et al. (2023)	Prospective cohort	UC patients with high vs. low anxiety/depression-related scale scores	Taxa and diversity	Higher psychological symptom burden was mainly associated with taxonomic alterations involving Enterobacteriaceae, Sutterella, Veillonella, Clostridiaceae, Klebsiella, Enterococcaceae, Gemellaceae, Streptococcus, and other taxa. Shannon and Chao diversity indices showed no significant differences across UC disease-activity subgroups.	Higher anxiety/depression-related scores	MANCOVA adjusted for age and disease activity reported significant taxon-level differences. HADS-A was associated with increased TM7 (*P* = 0.035), Gemellaceae (*P* < 0.001), Sutterella (*P* = 0.003), and Veillonella (*P* = 0.008), and decreased Clostridiaceae (*P* = 0.014) and Klebsiella (*P* = 0.013). HADS-D was associated with increased Enterococcaceae (*P* = 0.039), Gemellaceae (*P* = 0.008), Sutterella (*P* < 0.001), and Veillonella (*P* < 0.001), and decreased Clostridiaceae (*P* < 0.001). Effect sizes/CIs were not uniformly reported.	Supports an association between taxonomic dysbiosis and psychological symptoms; alpha-diversity evidence was not significant
Humbel et al. (2020)	Prospective cohort	IBD patients in remission with higher vs. lower psychological distress	Diversity and taxa	Higher perceived stress was associated with lower alpha diversity; anxiety/depression were associated with beta-diversity differences and reduced abundance of multiple taxa, including Lachnospiraceae, Ruminococcaceae, Veillonellaceae, Clostridia, Bacilli, and Bacteroidia.	Higher anxiety, depression, perceived stress, and impaired QoL	Higher perceived stress was associated with reduced Shannon/Simpson diversity; anxiety/depression showed significant beta-diversity clustering. Taxon-level associations were reported after BH correction, including Bifidobacterium in CD and Desulfovibrio in UC for depression (*q* < 0.05). Exact effect sizes/CIs were not uniformly reported.	Supports microbiota–psychological associations in remission-stage IBD
Feng et al. (2022)	Prospective cohort	CD patients with psychological disorders vs. CD patients without psychological disorders and healthy controls	Diversity, taxa, and bile acid metabolism	CD patients, particularly those with psychological disorders, showed reduced fecal microbiota alpha diversity. CD patients with psychological disorders showed enrichment of Enterobacteriaceae, Clostridiales, and Ruminococcus gnavus. Gut microbiota dysbiosis was linked to altered bile acid metabolism, including reduced secondary bile acids and increased serum 7-DHCA.	Higher SAS/SDS scores; anxiety and/or depressive symptoms	Chao1 and Shannon alpha diversity were significantly lower in CD than HC, and lower in CD patients with psychological disorders than those without psychological disorders. Bray–Curtis PCoA showed significant compositional separation between CD and HC (PERMANOVA *P* < 0.0001). LEfSe identified taxa enriched in CD-PD, including Enterobacteriaceae, Clostridiales, and R. gnavus. Fecal TDCA, TLCA, and TβMCA positively correlated with SAS, fecal 12-DHCA and HDCA negatively correlated with SDS, and serum 7-DHCA positively correlated with SDS. Exact effect sizes/CIs were not uniformly reported.	Supports associations among gut dysbiosis, bile acid alteration, and psychological symptoms in CD
Yuan et al. (2021)	Prospective observational study	Active UC patients with depression/anxiety vs. active UC patients without depression/anxiety, non-IBD patients with depression/anxiety, and healthy controls	Diversity, taxa, metabolomics, and proteomics	Active UC patients with depression/anxiety showed lower fecal microbial richness and diversity, with increased Lactobacillales, Sellimonas, Streptococcus, Enterococcus, and decreased Prevotella_9 and Lachnospira. Multi-omics analyses also showed altered serum metabolites, including increased glycocholic acid/glycochenodeoxycholate and decreased 2′-deoxy-D-ribose and L-pipecolic acid, together with reduced immunoglobulin proteins.	Higher PHQ-9/GAD-7 scores; depression and anxiety in active UC	UC depression/anxiety groups showed lower Shannon and PD whole-tree diversity, especially in Phase 1 (*P* = 0.07 for depression; *P* < 0.05 for anxiety). Depression level had a moderate impact on gut microbiota structure by PERMANOVA/Adonis (*R*^2^ > 0.015). Sellimonas, Lactobacillales, and Bacilli were significantly enriched after multiple testing correction (*P* < 6.3 × 10^−5^). Metabolites and proteins associated with UCD/UCA were identified by Student’s *t*-test, general linear regression, and pathway analyses. Exact effect sizes/CIs were not uniformly reported.	Supports multi-omics associations among gut dysbiosis, metabolic/proteomic alterations, and depression/anxiety in active UC
Thomann et al. (2022)	Prospective cohort	Active IBD patients with varying depression and fatigue severity	Taxa and functional metagenomics	Depression and fatigue were associated with specific taxonomic–functional microbiome patterns rather than alpha-diversity changes. Depression was linked to lower abundance of SCFA-producing genera, including Odoribacter, Alistipes, and Anaerotruncus, and to glycan/pectin/carbohydrate metabolism modules. Fatigue was linked to Intestinimonas, Anaerotruncus, Eubacterium, Clostridiales g.i.s., and amino acid/carbohydrate metabolism modules.	Higher HADS-D depression scores and WEIMuS fatigue scores	Bayesian network/triangular motif analysis identified depression-related genera and functional modules with moderate-to-strong evidence, including Odoribacter–depression (Log10BF10 = 1.956; ρ = −0.457) and Odoribacter–dermatan sulfate degradation (Log10BF10 = 2.611; ρ = 0.498). Fatigue-related motifs included Clostridiales g.i.s.–pentose phosphate pathway (Log10BF10 = 2.900; ρ = −0.517). Neither depression nor fatigue was associated with Shannon alpha diversity or inflammatory markers. Effect sizes/CIs were not uniformly reported.	Supports functional microbiome associations with depression/fatigue in active IBD; alpha-diversity evidence was not significant
Chen et al. (2021)	Pilot observational study	UC patients with depression vs. UC patients without depression and healthy controls	Diversity and taxa	UC patients with depression showed the lowest microbial abundance and diversity. Compared with UC patients without depression and healthy controls, the UC depression group showed decreased Firmicutes, Clostridia, Clostridiales, Lachnospiraceae, Prevotellaceae, and Roseburia, and increased Proteobacteria, Gammaproteobacteria, Bacilli, Enterobacteriales, Lactobacillales, Ruminococcaceae, Enterobacteriaceae, Escherichia-Shigella, and Enterococcus.	Depression assessed by SDS	UC patients with depression had more severe UC disease activity than those without depression (*P* = 0.006). Pairwise comparisons showed significant differences in Sobs, Ace, Shannon, and Chao indices among groups, with the lowest values in UC patients with depression. Firmicutes, Clostridia, and Clostridiales were lower in UC depression (*P* < 0.001), whereas Proteobacteria, Gammaproteobacteria, Bacilli, Enterobacteriales, Ruminococcaceae, Enterobacteriaceae, Escherichia-Shigella, and Enterococcus were higher (mostly *P* < 0.05 to *P* < 0.01). Exact effect sizes/CIs were not reported.	Supports an association between depression in UC and reduced microbial diversity with taxonomic dysbiosis
Liu et al. (2025)	Randomized controlled trial	Mild-to-moderate UC patients with anxiety and/or depression receiving probiotic LAB plus mesalazine vs. mesalazine alone for 8 weeks	Probiotic intervention and microbiota composition	Probiotic LAB supplementation increased Firmicutes abundance, improved GMHI, reduced MDI, increased Gram-positive phenotype, reduced potentially pathogenic phenotype, and increased the Firmicutes/Bacteroidota ratio. Genus-level changes were not statistically significant, although higher SCFA-producing taxa such as Blautia and Veillonella and lower pathobionts such as Bacteroides and Escherichia-Shigella were observed.	Improvement in HADS-anxiety and HADS-depression scores; UC disease activity assessed by MMS and MES	Anxiety improved in 65.4% of the probiotic group vs. 34.6% of controls (RR = 1.89, 95% CI: 1.07–3.54, *P* = 0.03). Depression improvement was higher in the probiotic group but not significant (53.8% vs. 30.8%; RR = 1.75, 95% CI: 0.92–3.52, *P* = 0.09). Anxiety scores decreased in both groups, and MES decreased significantly only in the probiotic group (1.62 ± 0.50 to 1.27 ± 0.53; *P* = 0.02). Firmicutes/Bacteroidota ratio increased significantly after LAB (*P* = 0.04).	Supports probiotic-associated improvement in anxiety and gut microbiota composition in UC with emotional disturbance; depression improvement was not statistically significant
K et al. (2024)	Randomized controlled trial	CD patients receiving COBMINDEX intervention vs. wait-list control for 3 months	Behavioral intervention and microbiota composition	COBMINDEX was associated with taxonomic abundance changes but not significant alpha- or beta-diversity changes. Significant changes occurred only in the COBMINDEX group, including increased Deferribacteres and Mucispirillum and decreased Streptococcaceae, Coriobacteriaceae, and Lachnospiraceae. Several altered taxa correlated with psychological distress and inflammatory markers.	Psychological distress and QoL measures, including SIBDQ, PSS4, GSI, SF-12, and HBI-related outcomes	At baseline, microbial taxa correlated with psychological markers: Lachnospiraceae with PSS4 (*r* = −0.37, *P* = 0.01), Subdoligranulum with SIBDQ (*r* = 0.35, *P* = 0.02) and PSS4 (*r* = −0.351, *P* = 0.01), and Enterococcus with GSI (*r* = 0.38, *P* = 0.01). After COBMINDEX, significant taxonomic changes included increased Deferribacteres (LDA = 3.51, *P* = 0.038) and Mucispirillum (LDA = 3.43, *P* = 0.038), and decreased Streptococcaceae (LDA = 3.63, *P* = 0.038), Coriobacteriaceae (LDA = 3.17, *P* = 0.043), and Lachnospiraceae (LDA = 3.08, *P* = 0.019). No significant changes in alpha or beta diversity were observed. Effect sizes/CIs for psychological outcomes were not uniformly reported in this microbiome analysis.	Supports associations between behavioral intervention, taxonomic microbiota changes, psychological distress, and inflammation in CD; diversity evidence was not significant
Ewais et al. (2021)	Pilot randomized controlled trial	Adolescents and young adults with IBD and depression receiving MBCT vs. treatment as usual	Mindfulness intervention and microbiome diversity	Metagenomic sequencing showed low Shannon diversity at baseline and a non-significant increase in diversity in both MBCT and TAU groups after 8 weeks. Microbiome analysis was exploratory and limited by substantial attrition.	Depression, anxiety, stress, coping, mindfulness, and IBD-related QoL	ITT analysis showed significantly lower depression at 8 weeks in the MBCT group vs. TAU (mean difference Δ = −6.0, 95% CI: −10.8 to −1.2, *P* = 0.015) and lower stress (Δ = −5.1, 95% CI: −10.1 to −0.0, *P* = 0.049). Per-protocol analysis similarly showed lower depression (Δ = −6.3, 95% CI: −11.4 to −1.2, *P* = 0.015) and stress (Δ = −6.0, 95% CI: −11.5 to −0.5, *P* = 0.032). Shannon diversity showed only a non-significant increase after 8 weeks; microbiome data were available for 19 participants.	Supports feasibility and psychological benefit of MBCT in youth with IBD; microbiome diversity evidence was exploratory and not statistically significant
Shang et al. (2023)	Prospective controlled clinical study	Male and female UC patients receiving same-sex donor FMT vs. placebo	FMT intervention, diversity, taxa, and inflammation	Same-sex donor FMT increased gut microbiota abundance and diversity in both male and female UC patients. In male patients, FMT reduced Clostridia, Staphylococcaceae, Megamonas, Romboutsia, and Desulfovibrionaceae, and increased Prevotella, Lactobacillus, and Bifidobacterium. In female patients, FMT reduced Escherichia-Shigella, Desulfovibrionaceae, Erysipelotrichaceae, Veillonella, and Staphylococcaceae, and increased Porphyromonadaceae, Prevotella, Lactobacillus, Bifidobacterium, Akkermansia, and Coprococcus.	SAS and SDS scores; UC clinical symptoms and Mayo score	FMT significantly reduced diarrhea, abdominal pain, bloody stool, mucosal lesion, and Mayo scores compared with baseline and placebo groups (mostly *P* < 0.05). SDS and SAS scores decreased significantly after FMT in both male and female patients, with stronger reductions over 9 weeks (mostly *P* < 0.001 to *P* < 0.0001 vs. baseline; significant vs. placebo). hsCRP decreased after FMT in male patients at 9 weeks and in female patients from 3 weeks onward. Shannon, Simpson, ACE, Chao1, PCA, and PCoA analyses indicated increased alpha diversity and altered overall microbiota structure after FMT, whereas placebo groups showed no major changes. Exact effect sizes/CIs were not reported.	Supports FMT-associated improvement in UC symptoms, psychological scores, inflammation, and microbiota diversity/composition; causal interpretation remains limited by study design

IBD, inflammatory bowel disease; UC, ulcerative colitis; CD, Crohn’s disease; RCT, randomized controlled trial; SAS, Self-Rating Anxiety Scale; SDS, Self-Rating Depression Scale; HADS, Hospital Anxiety and Depression Scale; PHQ-9, Patient Health Questionnaire-9; GAD-7, Generalized Anxiety Disorder-7; DASS, Depression Anxiety Stress Scales; QoL, quality of life; SCFA, short-chain fatty acid; FMT, fecal microbiota transplantation; LAB, lactic acid bacteria; GMHI, Gut Microbiome Health Index; MDI, Microbial Dysbiosis Index; 7-DHCA, 7-dehydrocholic acid; RR, relative risk; CI, confidence interval; LDA, linear discriminant analysis; PERMANOVA, permutational multivariate analysis of variance. Because the included studies differed in microbiota profiling methods, psychological assessment tools, statistical models, and reporting formats, exact *p*-values, effect sizes, and confidence intervals were not consistently available for all study–outcome pairs. Therefore, this table summarizes the direction, statistical reporting, and interpretive strength of the available evidence rather than providing pooled quantitative estimates.

### 3.2 Findings from cohort studies

#### 3.2.1 Microbial α-diversity and psychological symptoms

Across multiple cohort studies, reduced gut microbial α-diversity was associated with anxiety and depressive symptoms in patients with IBD. Scaldaferri et al. (2023) reported lower Shannon and Chao indices in UC patients with elevated anxiety or depression scores. These findings indicated reduced microbial richness and evenness. Similarly, Chen et al. (2021) observed significantly lower microbial diversity in UC patients with depression than in those without depression. Feng et al. (2022) reported comparable findings in CD patients with psychological symptoms.

However, not all studies showed consistent results. Thomann et al. (2022) found no significant association between α-diversity and depression or fatigue. They also found no significant association with inflammatory markers, although microbial functional profiles were clearly altered.

#### 3.2.2 Microbial taxa associated with psychological symptoms

Distinct microbial signatures were observed in IBD patients with anxiety or depression. In UC, Scaldaferri et al. (2023) reported increased abundance of pro-inflammatory and opportunistic taxa. These included Enterobacteriaceae, Streptococcus, Veillonella, Klebsiella, and Clostridiaceae. In contrast, butyrate-producing bacteria, such as Ruminococcaceae and Roseburia, were reduced.

Similarly, Chen et al. (2021) found increased Proteobacteria, Enterobacteriaceae, and Escherichia–Shigella in depressed UC patients. These changes were accompanied by decreased Firmicutes, Clostridia, and Roseburia.

In CD, Feng et al. (2022) reported a positive correlation between Ruminococcus gnavus abundance and anxiety/depression scores. They also observed depletion of beneficial taxa, including Faecalibacterium prausnitzii. Yuan et al. (2021) further identified disease-specific associations. Depression in CD was linked to increased Bifidobacterium, whereas anxiety in UC was associated with elevated Desulfovibrio.

#### 3.2.3 Functional alterations in the microbiome

Functional analyses suggested that psychological symptoms were associated with changes in microbial metabolic potential. Thomann et al. (2022) reported that depression was linked to reduced abundance of Odoribacter species involved in pectin degradation. Fatigue was associated with changes in amino acid metabolism and the pentose phosphate pathway.

Feng et al. (2022) further identified associations between anxiety/depression and disrupted bile acid metabolism. These changes included reduced secondary bile acids and elevated serum 7-dehydrocholic acid in CD patients.

Using a multi-omics approach, Yuan et al. (2021) showed that alterations in metabolites and immunoglobulin profiles were associated with psychological symptoms in patients with active UC.

#### 3.2.4 Independence from inflammatory activity

Several cohort studies attempted to distinguish microbiota–psychological associations from the effects of intestinal inflammatory activity. Humbel et al. (2020) examined patients with IBD in clinical remission. They found that psychological distress remained associated with gut microbiota composition despite the absence of clinically active disease. These associations included diversity-related and taxonomic differences. This finding suggests that microbiota–psychological associations may not be solely attributable to overt intestinal inflammation.

Similarly, Thomann et al. (2022) reported that depression- and fatigue-related microbiome alterations were not consistently associated with inflammatory markers. Although no significant association with α-diversity was observed, depression was linked to specific microbial taxa and functional pathways. These included reduced abundance of Odoribacter, Alistipes, and Anaerotruncus, as well as altered carbohydrate-related metabolic functions. These results indicate that functional microbiome alterations may be related to psychological symptoms through mechanisms that are partly independent of conventional inflammatory activity.

Other studies also provided indirect support for this interpretation. Feng et al. (2022) identified concurrent alterations in gut microbiota and bile acid metabolism in CD patients with psychological disorders. Thomann et al. (2022) reported multi-omics associations involving gut microbiota, metabolites, and immunoglobulin profiles in active UC patients with anxiety and depression. These findings suggest that metabolic and immune-related pathways may contribute to psychological symptoms beyond a simple reflection of disease activity.

Nevertheless, this evidence should be interpreted cautiously. The included studies differed in disease activity definitions, inflammatory markers, adjustment models, and microbiota profiling methods. Therefore, the current evidence supports partial, rather than complete, independence of microbiota–psychological associations from inflammatory activity.

### 3.3 Findings from randomized controlled trials

Four RCTs met the inclusion criteria, evaluating probiotics, FMT, and psychological interventions with concurrent microbiota assessment (Ewais et al., 2021; K et al., 2024; Liu et al., 2025; Shang et al., 2023). All studies reported both microbiota and psychological outcomes.

#### 3.3.1 Probiotic intervention

Liu et al. (2025) conducted an 8-week RCT in 52 patients with mild-to-moderate UC. The study compared lactic acid bacteria (LAB) plus standard therapy with standard therapy alone. Probiotic supplementation significantly improved anxiety and depression scores. It also reduced Mayo endoscopic scores.

Microbiota analysis showed increased α-diversity and a higher Firmicutes/Bacteroidota ratio. It also showed enrichment of short-chain fatty acid (SCFA)-producing bacteria and reduction of pathogenic taxa. These microbial changes were associated with improvements in both disease activity and psychological outcomes. This suggests a potential mechanistic link.

#### 3.3.2 Psychological interventions with microbiota assessment

Two RCTs evaluated psychological interventions alongside microbiota profiling. K et al. (2024) conducted a 3-month intervention in 49 patients with mild-to-moderate CD. The intervention combined cognitive behavioral therapy, mindfulness, and daily physical activity. It reduced depression and stress levels and improved quality of life.

Microbiota analysis showed increased Deferribacteres and decreased Streptococcaceae, Coriobacteriaceae, and Lachnospiraceae. Baseline Firmicutes abundance was also correlated with psychological measures.

Ewais et al. (2021) evaluated an 8-week mindfulness-based cognitive therapy (MBCT) program in 64 young patients with IBD. MBCT significantly reduced depression and stress. It also improved adaptive coping and mindfulness. Although both groups showed non-significant increases in microbial diversity, high attrition rates limited interpretation. The authors concluded that MBCT was feasible and beneficial for psychological outcomes. However, larger studies are needed to clarify microbiota-mediated mechanisms.

#### 3.3.3 FMT

Shang et al. (2023) evaluated a 9-week FMT intervention in 136 patients with active UC. Compared with placebo, FMT significantly increased microbial richness and diversity. Anxiety and depression scores were also significantly reduced after the intervention. More pronounced improvements were observed in female patients.

Microbial changes showed sex-specific patterns. In males, Clostridiales and Desulfovibrionaceae decreased, whereas Prevotella, Lactobacillus, and Bifidobacterium increased. In females, Escherichia–Shigella and Desulfovibrionaceae decreased, whereas Porphyromonadaceae, Prevotella, Lactobacillus, and Bifidobacterium increased.

### 3.4 Risk of bias assessment

Risk of bias was assessed using the CASP checklists for cohort studies and randomized controlled trials. Overall, the included studies clearly defined their research questions. They were considered to have acceptable methodological quality. However, several domain-specific concerns were identified.

In cohort studies, the main limitations included incomplete adjustment for confounders, heterogeneous microbiota assessment methods, variable psychological outcome measurements, and limited external validity. In randomized controlled trials, the main concerns included incomplete reporting of allocation concealment or blinding, small sample sizes, short follow-up durations, attrition in biological sample collection, and limited precision of outcome estimates.

These issues may have introduced residual confounding, measurement bias, performance bias, detection bias, and reduced comparability across studies. Overall, the included studies were judged to be of moderate methodological quality. Therefore, the findings should be interpreted cautiously. Detailed domain-level CASP assessment results are provided in Supplementary material.

## 4 Discussion

### 4.1 Principal findings

By integrating evidence from both prospective cohort studies and randomized controlled trials, this review identifies a consistent pattern suggesting that anxiety and depressive symptoms in patients with IBD are associated with gut microbiota dysbiosis.

At the observational level, cohort studies consistently identified a characteristic microbial pattern associated with psychological symptoms. This pattern included reduced α-diversity, enrichment of pro-inflammatory taxa, and depletion of SCFA-producing bacteria. It was observed across different IBD subtypes and study populations. This suggests that it may represent a shared microbial signature related to psychological comorbidities in IBD.

Notably, in several studies, these associations persisted after adjustment for inflammatory markers. They were also observed when analyses were restricted to patients in remission. These findings argue against a purely secondary effect of intestinal inflammation. Instead, they suggest that microbiota–psychological interactions may reflect a relatively independent dimension of gut–brain axis dysregulation.

At the interventional level, evidence from RCTs provides further support for a potential mechanistic link. Microbiota-targeted strategies, including probiotics and fecal microbiota transplantation, were associated with concurrent improvements in psychological symptoms and partial restoration of microbial composition or function. This parallel change suggests that gut microbiota may mediate mental health regulation in IBD.

However, the effects were not uniform across interventions. Microbiota-targeted therapies tended to produce concordant changes in microbial and psychological outcomes. In contrast, some psychological interventions significantly improved mental health without causing substantial microbiota alterations. This divergence suggests that multiple and partly independent pathways may contribute to psychological symptom modulation in IBD. Their relative contributions may vary according to intervention type.

Recent studies outside the IBD context have also linked gut microbiota dysbiosis to psychiatric disorders. These include depression, anxiety, and cognitive impairment. Such findings support the broader relevance of the microbiota–gut–brain axis (Huang et al., 2025; Wu et al., 2026a). Recent microbiome and neuroimaging evidence in first-episode major depressive disorder has further linked altered gut microbial profiles to changes in functional brain connectivity (Wu et al., 2026b). This suggests that microbial alterations may participate in psychological and neurobiological dysfunction beyond intestinal inflammation.

In parallel, depression and anxiety have often been associated with elevated peripheral inflammatory markers. This indicates that microbiota-related, inflammatory, and psychological pathways may overlap. However, they are not necessarily identical. Therefore, in IBD, microbiota–psychological associations should be interpreted as partially independent of inflammatory activity, rather than completely separate from it.

Taken together, current evidence suggests that gut microbiota alterations in IBD may be more than passive correlates of psychological comorbidities. However, most available evidence is observational or derived from small-scale interventional studies. Therefore, the directionality, causal relationships, and dominant pathways linking microbiota alterations to psychological symptoms remain unclear.

### 4.2 Mechanistic insights

The gut–brain axis is a multi-layered communication network. It links the gut microbiota, immune system, and central nervous system (Morton et al., 2023). In IBD-related anxiety and depression, the available clinical evidence does not support a single linear mechanism. Instead, the findings of this review suggest that microbiota–psychological associations may involve overlapping immune–inflammatory, microbial metabolic, and neuroendocrine pathways. However, the strength of evidence differs across these mechanisms.

Based on the included studies, the most consistently supported mechanisms involve reduced microbial diversity, enrichment of pro-inflammatory or opportunistic taxa, depletion of SCFA-producing bacteria, and alterations in bile acid metabolism. In contrast, other mechanisms are mainly supported by indirect evidence in the IBD population. These include tryptophan metabolism, vagal signaling, HPA-axis activation, microglial responses, and neuroendocrine regulation. Therefore, the following discussion prioritizes mechanisms with direct support from the included clinical studies. More speculative pathways are presented as complementary hypotheses.

Within the immune–inflammatory axis, several included studies reported enrichment of pro-inflammatory or opportunistic taxa in IBD patients with psychological symptoms. These taxa included Enterobacteriaceae, Escherichia–Shigella, Streptococcus, Veillonella, and Ruminococcus gnavus. Such microbial patterns may promote inflammatory signaling through microbial components such as lipopolysaccharide. Lipopolysaccharide can activate TLR4-mediated NF-κB signaling and promote the release of pro-inflammatory cytokines. These cytokines include interleukin-6, tumor necrosis factor-α, and interleukin-1β (Ge et al., 2022).

These inflammatory mediators may influence central nervous system function through systemic circulation, immune–neural communication, or vagal pathways. In this way, they may contribute to anxiety- and depression-like symptoms (Fakhfouri et al., 2024; Sălcudean et al., 2025). Clinical observations that psychological symptoms may improve in parallel with anti-inflammatory treatment further support the relevance of this pathway (Chang et al., 2024). However, associations between inflammatory markers and psychological symptoms were not consistent across the included studies. This suggests that inflammation is an important contributor, but not the only pathway linking microbiota alterations to psychological symptoms in IBD.

The microbial metabolic axis appears to be one of the most directly supported mechanisms in this review. Across several cohort studies, psychological symptoms were associated with depletion of SCFA-producing or butyrate-associated bacteria. These included Roseburia, Faecalibacterium prausnitzii, Lachnospiraceae, and Ruminococcaceae.

SCFAs can regulate immune cell function, intestinal barrier integrity, blood–brain barrier homeostasis, and neuronal energy metabolism. These effects may involve histone deacetylase inhibition and G protein-coupled receptor signaling (Pi et al., 2024; Sun et al., 2024). Therefore, reduced SCFA-producing capacity may provide a plausible metabolic route linking gut dysbiosis to psychological symptoms.

Importantly, this pathway may also help explain why microbiota–psychological associations persist in some patients even when conventional inflammatory activity is not prominent. Nevertheless, not all studies have reported consistent associations between SCFAs and psychological outcomes. The magnitude and direction of these effects may depend on baseline microbiota composition, host metabolic context, and methodological variability (Scaldaferri et al., 2023).

Bile acid metabolism represents another relatively well-supported metabolic pathway. Feng et al. identified concurrent gut microbiota dysbiosis and bile acid metabolism alterations in CD patients with psychological disorders. These alterations included reduced secondary bile acids and increased serum 7-dehydrocholic acid.

These findings suggest that altered microbial metabolism may influence psychological symptoms through bile acid-related signaling, in addition to SCFAs. Bile acids can interact with immune, metabolic, and neural pathways. Therefore, this mechanism may be relevant for linking microbial functional changes with gut–brain communication (Pan et al., 2024; Xu and Lu, 2025). However, evidence for this pathway remains limited to a small number of studies. Larger cohorts are needed for validation.

Other microbial metabolic pathways may also contribute to microbiota–brain communication. These include tryptophan metabolism and indole derivative production. Under inflammatory conditions, tryptophan metabolism can shift toward the kynurenine pathway and generate neuroactive metabolites. Gut microbiota may also influence serotonin-related signaling (Chen et al., 2024; Zhang et al., 2025). In addition, indole derivatives may affect central nervous system function through immune, metabolic, or barrier-related pathways (Meng, 2026).

These mechanisms are biologically plausible and have been widely discussed in the broader gut–brain axis literature. However, direct clinical evidence linking tryptophan metabolism or indole derivatives to anxiety or depression in IBD remains limited in the studies included in this review. Therefore, these pathways should be interpreted as complementary hypotheses rather than established mechanisms.

Neuroendocrine and neural pathways may provide additional explanations for the possible bidirectionality of microbiota–psychological associations. Psychological stress can activate the HPA axis and the sympathetic nervous system. These responses may alter inflammatory activity, intestinal permeability, mucosal immune responses, antimicrobial peptide secretion, gut motility, luminal conditions, and microbial community structure (Duan et al., 2021; Madison and Bailey, 2024; Marwaha et al., 2025).

Stress-induced neuroimmune alterations may further reinforce central inflammatory signaling. These alterations include microglial metabolic reprogramming and changes in synaptic plasticity (Fang et al., 2025; Huang et al., 2024). The vagus nerve may also serve as a communication route between the gut and the brain. Preclinical studies suggest that some probiotic-related anxiolytic effects may depend on intact vagal signaling (Verma et al., 2024).

Central nervous system activity and chronic stress may also modulate gut motility, secretion, and immune responses through vagal and sympathetic pathways. These changes may reshape microbial ecology (Zou et al., 2024). However, direct clinical evidence for vagal or HPA-axis-mediated mechanisms in IBD-related anxiety and depression remains limited. Compared with microbial diversity, SCFA-producing bacteria, and bile acid metabolism, these pathways are less well established. Therefore, they should be viewed as biologically plausible routes rather than mechanisms directly confirmed by the included studies.

Stress-related changes may also influence microbial metabolic function. Under chronic stress, beneficial taxa may decrease, SCFA production may be reduced, and intestinal barrier integrity may be impaired. These changes may contribute to the persistence or worsening of psychological symptoms (Jia et al., 2025). In addition, stress-induced changes in luminal pH and redox conditions may selectively suppress butyrate-producing bacteria. This may weaken the protective effects of microbial metabolic pathways (Kalkan et al., 2025). These findings provide a plausible explanation for feedback between psychological stress, microbial ecology, and metabolic disruption. However, direct evidence in IBD-related anxiety and depression remains limited.

Taken together, the current evidence most strongly supports a framework centered on gut dysbiosis, enrichment of pro-inflammatory or opportunistic taxa, depletion of SCFA-producing bacteria, and microbial metabolic disruption, especially bile acid alteration. Neuroendocrine, vagal, tryptophan-related, indole-related, and microglial pathways remain biologically plausible mechanisms. However, their direct contribution to psychological symptoms in IBD requires further validation.

Although these pathways provide a coherent explanation for microbiota–psychological interactions, they should be interpreted as mechanistic hypotheses rather than established causal pathways. The current evidence base is still largely derived from observational studies and small-scale clinical trials. This limits causal inference.

Emerging evidence from humanized models suggests that restoration of SCFA production or modulation of tryptophan metabolism may ameliorate stress-related behaviors. These effects may be achieved through specific probiotics or fecal microbiota transplantation (Kalkan et al., 2025). Future studies should integrate longitudinal cohort designs, standardized microbiome profiling, metabolomics, immune phenotyping, formal mediation analyses, and neuroimaging techniques. Such studies are needed to clarify temporal relationships, identify dominant pathways, and determine whether specific microbiota-related changes contribute causally to psychological comorbidities in IBD.

### 4.3 Clinical implications

Although current evidence shows a close association between gut microbiota alterations and anxiety and depressive symptoms in patients with IBD, this evidence has not yet translated into microbiota-based clinical decision-making. This association appears to be partly independent of inflammatory activity. However, its clinical application remains limited.

At present, IBD management is still mainly focused on controlling inflammation. Psychological comorbidities are usually managed symptomatically. Microbiota–immune–neurobiological interactions are rarely integrated into routine care. This inflammation-centered model may be insufficient for some patients. This is especially relevant for patients whose intestinal inflammation is well controlled but who continue to experience psychological symptoms. In these patients, non-inflammatory regulatory pathways may contribute to persistent psychological burden.

From a translational perspective, clinically actionable findings should be distinguished from investigational implications. Currently, the clinically actionable aspects mainly include routine recognition and assessment of anxiety and depressive symptoms in patients with IBD. Psychological comorbidities should also be considered when evaluating overall disease burden. In addition, psychological or behavioral support should be integrated into multidisciplinary care.

In contrast, microbiota-based clinical applications remain investigational. These include the use of microbiota profiles to stratify patients, predict psychological symptoms, or guide treatment selection. Similarly, microbiota-targeted interventions should not yet be considered routine treatments for anxiety or depression in IBD. These interventions include probiotics, prebiotics, SCFA supplementation, and fecal microbiota transplantation. Their efficacy, optimal target populations, dosing strategies, durability, and safety require further validation.

From both phenotypic and mechanistic perspectives, psychological comorbidities in IBD are unlikely to represent a uniform clinical entity. Rather, they may reflect different combinations of dominant pathways. In some patients, psychological symptoms may persist despite adequate inflammatory control. This may suggest a greater contribution of microbial metabolic and neuroregulatory mechanisms.

In other patients, especially those with poor response to anti-inflammatory therapy and pronounced dysbiosis, microbial composition and metabolite profiles may play a more central role. Patients with fluctuating symptoms and prominent stress-related features may show coupled dysregulation across neural, immune, and microbial pathways. However, these proposed subgroups remain hypothetical. They require prospective validation before being used in clinical decision-making.

Emerging interventional evidence provides preliminary support for microbiota- and neuroregulation-informed approaches. Microbiota-targeted strategies have been associated with improvements in psychological outcomes and changes in microbial composition or metabolic function. These strategies include probiotics, SCFA supplementation, and fecal microbiota transplantation. Their effects may occur through immune or metabolic pathways.

In contrast, psychological and behavioral interventions may act mainly through neuroregulatory mechanisms. They may also indirectly influence the gut environment. However, the comparative efficacy, optimal timing, dose–response relationships, and target populations of these interventions remain uncertain.

For example, fecal microbiota transplantation may warrant further study in patients with marked dysbiosis because it can induce large-scale microbial restructuring. Probiotics or prebiotics may be more suitable for evaluation in patients with mild-to-moderate symptoms or during maintenance phases. Interventions targeting neural regulation may be particularly relevant for individuals with stress-dominant or affective dysregulation profiles. However, these possibilities require validation in dedicated clinical studies.

From a clinical decision-making perspective, microbiota modulation should be considered a complementary strategy if future evidence supports its use. It should not be regarded as a replacement for anti-inflammatory therapy. Future studies should examine whether stratified assessment can support pathway-matched interventions. Such assessment may integrate inflammatory status, psychological symptom profiles, and microbiota characteristics. This approach may improve treatment precision in the future. However, its clinical utility requires prospective validation.

Future research should bridge the gap between associative evidence and clinical application. Large-scale longitudinal studies are needed to clarify the temporal and causal relationships between microbiota alterations and psychological symptoms. Standardized microbiome profiling techniques and psychological assessment tools are also essential. This would improve comparability across studies.

In addition, randomized controlled trials should systematically evaluate different interventions. Key issues include comparative efficacy, optimal timing, dose–response relationships, long-term durability, and safety. Integrative multi-omics approaches may help identify patient subgroups most likely to benefit from microbiota-targeted therapies. These approaches may include metagenomics, metabolomics, and immune phenotyping. They may also support the development of precision medicine in this field.

Overall, current clinical practice should focus on early identification of psychological comorbidities, integrated psychosocial care, and optimized IBD disease control. Microbiota-based stratification and microbiota-targeted therapies for psychological symptoms should remain within the research setting. Larger and more standardized trials are needed before routine clinical use can be recommended. Given that current evidence is mainly derived from associative analyses and small-scale interventional studies, clinical implementation of microbiota-based therapies remains premature. Their true efficacy, safety, durability, and appropriate clinical indications require further validation in high-quality studies.

### 4.4 Strengths and limitations

This review has several notable strengths. First, it integrates evidence from both prospective cohort studies and randomized controlled trials, allowing for a comprehensive evaluation of the role of the gut microbiota in psychological comorbidities in IBD from both associative and interventional perspectives. Compared with previous reviews that primarily focused on a single study design, this approach enables a more coherent interpretation of potential causal pathways by linking temporal associations with intervention effects.

Second, this study highlights that microbiota–psychological associations may be at least partially independent of inflammatory activity. This observation provides an important conceptual advance. It suggests that microbial metabolic and neuroregulatory mechanisms may contribute to psychological comorbidities in IBD beyond inflammatory pathways. These mechanisms may not be fully captured by conventional inflammatory markers. This perspective supports the development of mechanism-based stratification and more targeted therapeutic strategies.

Several limitations should also be acknowledged. First, substantial heterogeneity existed across the included studies. The studies differed in patient characteristics, IBD subtype, disease activity status, microbiome profiling methods, psychological assessment tools, intervention types, comparator definitions, and follow-up durations. Microbiota outcomes were generated using different sequencing, profiling, and analytical approaches. Psychological symptoms were assessed using heterogeneous instruments with different constructs, thresholds, and scoring systems. The study populations also varied in IBD subtype, disease activity, age range, treatment background, and geographic setting.

Second, a meta-analysis was not performed because microbiota-related outcomes and statistical reporting formats varied substantially. The included studies reported heterogeneous microbiota outcomes. These included alpha diversity, beta diversity, specific taxa, microbiome health indices, microbial dysbiosis indices, bile acid profiles, functional pathways, metabolomic signatures, and proteomic features. Moreover, many studies did not provide standardized effect sizes, standard errors, confidence intervals, or sufficient raw data for recalculation. Therefore, reliable pooled estimates could not be generated. I^2^ statistics, subgroup analyses, sensitivity analyses, and publication bias assessment were also not performed. As a result, the conclusions of this review should be interpreted as a structured synthesis of current evidence rather than quantitatively pooled estimates.

Third, the evidence base remains limited. Only 10 studies were included, and several had relatively small sample sizes. This limited number of studies reduces the statistical robustness and generalizability of the findings. This is especially relevant for subgroup interpretation according to IBD subtype, disease activity status, intervention type, and psychological outcome. Therefore, the conclusions should be interpreted cautiously. They should not be generalized beyond the populations and settings represented in the included studies.

Fourth, several included studies did not adequately or consistently control for key potential confounders. These included diet and medication use, such as antibiotics, biologics, corticosteroids, and probiotic exposure. They also included lifestyle factors, such as physical activity, sleep quality, and stress. These variables may influence both gut microbiota composition and psychological outcomes, thereby introducing residual confounding. Therefore, the observed microbiota–psychological associations should be interpreted cautiously. Future studies should more systematically collect and adjust for these factors.

Furthermore, publication bias was not formally assessed. This was because the number and comparability of studies were insufficient for funnel plot-based or statistical evaluation. In addition, no standardized framework, such as GRADE, was applied to evaluate the overall certainty of evidence. This may affect the strength of the conclusions. Finally, only English-language studies were included, which may have introduced language and regional bias.

## 5 Conclusion

This systematic review suggests that gut microbiota dysbiosis is associated with anxiety and depressive symptoms in patients with IBD. This association may not be fully explained by inflammatory activity. The available evidence supports a mechanistic hypothesis involving immune–inflammatory, microbial metabolic, and neuroendocrine pathways. The relative contribution of these pathways may vary across disease stages and individuals. Clinically, these findings suggest that management of psychological comorbidities in IBD may need to extend beyond inflammation control and incorporate microbiota- and neuroregulation-informed strategies. However, current evidence remains limited, heterogeneous, and largely associative. Therefore, causal or bidirectional relationships cannot be firmly established. Further large-scale longitudinal studies, well-designed randomized controlled trials, and mechanistic investigations are needed to clarify directionality, test causal hypotheses, and guide precision interventions.

## Data Availability

The original contributions presented in this study are included in the article/Supplementary material, further inquiries can be directed to the corresponding authors.
