# Supplementary material for: Gut microbiota alterations associated with anxiety and depression in inflammatory bowel disease: a systematic review of prospective cohorts and randomized trials
Source: Front Microbiol. 2026 Jun 2;17:1853009. doi: 10.3389/fmicb.2026.1853009 (PMC13269098; doi:10.3389/fmicb.2026.1853009)
Supplement: Supplementary file 1 [file Supplementary_file_1.docx]

**SUPPLEMENTARY MATERIAL**

**Gut Microbiota and Anxiety/Depression in Inflammatory Bowel Disease: A Systematic Review**

**of Randomized Trials and Prospective Cohorts**

Tao Zhang^1^, Lin Wang^1^, Zhetan Ren^2^, Ru Man^1^, Xiaozhen Cheng^3^, Bowen Tang^1^, Jing Wang^4^, Xiumei Bu*^1^, Yongduo Yu*^4^

^1^Liaoning University of Traditional Chinese Medicine, Shenyang 110000, China.

^2^Department of general surgery, Beijing Shijitan Hospital, Capital Medical University, Beijing, 100000, China.

^3^Shenzhen Traditional Chinese Medicine Hospital, Shenzhen 518000, China.

^4^Second Affiliated Hospital, Liaoning University of Traditional Chinese Medicine, Shenyang 110000, China.

***Correspondence:**

**Corresponding Author1:**Xiumei Bu

**Email:**1366319241@qq.com

**Corresponding Author2:**Yongduo Yu

**Email:**yuyongduo@163.com

**Tao Zhang and Lin Wang contributed equally to this study.**

**Index of supplementary material**

Supplementary 1 PRISMA checklist 3

Supplementary 2 Search strategy 7

Supplementary 3 Risk assessment of prospective studies using CASP checklist. 9

Supplementary 4 Quality assessment of randomized Controlled Trials (RCT) using CASP checklist. 10

| **Section and Topic** | **Item #** | **Checklist item** | **Location where item is reported** |
| --- | --- | --- | --- |
| **TITLE** | | |  |
| Title | 1 | Identify the report as a systematic review. | 1 |
| **ABSTRACT** | | |  |
| Abstract | 2 | See the PRISMA 2020 for Abstracts checklist. | 2 |
| **INTRODUCTION** | | |  |
| Rationale | 3 | Describe the rationale for the review in the context of existing knowledge. | 2-3 |
| Objectives | 4 | Provide an explicit statement of the objective(s) or question(s) the review addresses. | 2-3 |
| **METHODS** | | |  |
| Eligibility criteria | 5 | Specify the inclusion and exclusion criteria for the review and how studies were grouped for the syntheses. | 1 |
| Information sources | 6 | Specify all databases, registers, websites, organisations, reference lists and other sources searched or consulted to identify studies. Specify the date when each source was last searched or consulted. | 3 |
| Search strategy | 7 | Present the full search strategies for all databases, registers and websites, including any filters and limits used. | 4 |
| Selection process | 8 | Specify the methods used to decide whether a study met the inclusion criteria of the review, including how many reviewers screened each record and each report retrieved, whether they worked independently, and if applicable, details of automation tools used in the process. | 4 |
| Data collection process | 9 | Specify the methods used to collect data from reports, including how many reviewers collected data from each report, whether they worked independently, any processes for obtaining or confirming data from study investigators, and if applicable, details of automation tools used in the process. | 4 |
| Data items | 10a | List and define all outcomes for which data were sought. Specify whether all results that were compatible with each outcome domain in each study were sought (e.g. for all measures, time points, analyses), and if not, the methods used to decide which results to collect. | 4 |
|  | 10b | List and define all other variables for which data were sought (e.g. participant and intervention characteristics, funding sources). Describe any assumptions made about any missing or unclear information. | 4 |
| Study risk of bias assessment | 11 | Specify the methods used to assess risk of bias in the included studies, including details of the tool(s) used, how many reviewers assessed each study and whether they worked independently, and if applicable, details of automation tools used in the process. | 4 |
| Effect measures | 12 | Specify for each outcome the effect measure(s) (e.g. risk ratio, mean difference) used in the synthesis or presentation of results. | 4 |
| Synthesis methods | 13a | Describe the processes used to decide which studies were eligible for each synthesis (e.g. tabulating the study intervention characteristics and comparing against the planned groups for each synthesis (item #5)). | 4 |
|  | 13b | Describe any methods required to prepare the data for presentation or synthesis, such as handling of missing summary statistics, or data conversions. | Not carried out |
|  | 13c | Describe any methods used to tabulate or visually display results of individual studies and syntheses. | 4 |
|  | 13d | Describe any methods used to synthesize results and provide a rationale for the choice(s). If meta-analysis was performed, describe the model(s), method(s) to identify the presence and extent of statistical heterogeneity, and software package(s) used. | 4 |
|  | 13e | Describe any methods used to explore possible causes of heterogeneity among study results (e.g. subgroup analysis, meta-regression). | Not carried out |
|  | 13f | Describe any sensitivity analyses conducted to assess robustness of the synthesized results. | Not carried out |
| Reporting bias assessment | 14 | Describe any methods used to assess risk of bias due to missing results in a synthesis (arising from reporting biases). | Not carried out |
| Certainty assessment | 15 | Describe any methods used to assess certainty (or confidence) in the body of evidence for an outcome. | Not carried out |
| **RESULTS** | | |  |
| Study selection | 16a | Describe the results of the search and selection process, from the number of records identified in the search to the number of studies included in the review, ideally using a flow diagram. | 5 |
|  | 16b | Cite studies that might appear to meet the inclusion criteria, but which were excluded, and explain why they were excluded. | Figure 1 |
| Study characteristics | 17 | Cite each included study and present its characteristics. | 5-6 |
| Risk of bias in studies | 18 | Present assessments of risk of bias for each included study. | 6 |
| Results of individual studies | 19 | For all outcomes, present, for each study: (a) summary statistics for each group (where appropriate) and (b) an effect estimate and its precision (e.g. confidence/credible interval), ideally using structured tables or plots. | 5-6 |
| Results of syntheses | 20a | For each synthesis, briefly summarise the characteristics and risk of bias among contributing studies. | 5-6 |
|  | 20b | Present results of all statistical syntheses conducted. If meta-analysis was done, present for each the summary estimate and its precision (e.g. confidence/credible interval) and measures of statistical heterogeneity. If comparing groups, describe the direction of the effect. | Not carried out |
|  | 20c | Present results of all investigations of possible causes of heterogeneity among study results. | Not carried out |
|  | 20d | Present results of all sensitivity analyses conducted to assess the robustness of the synthesized results. | Not carried out |
| Reporting biases | 21 | Present assessments of risk of bias due to missing results (arising from reporting biases) for each synthesis assessed. | Not carried out |
| Certainty of evidence | 22 | Present assessments of certainty (or confidence) in the body of evidence for each outcome assessed. | Not carried out |
| **DISCUSSION** | | |  |
| Discussion | 23a | Provide a general interpretation of the results in the context of other evidence. | 6-7 |
|  | 23b | Discuss any limitations of the evidence included in the review. | 7 |
|  | 23c | Discuss any limitations of the review processes used. | 7 |
|  | 23d | Discuss implications of the results for practice, policy, and future research. | 7 |
| **OTHER INFORMATION** | | |  |
| Registration and protocol | 24a | Provide registration information for the review, including register name and registration number, or state that the review was not registered. | 4 |
|  | 24b | Indicate where the review protocol can be accessed, or state that a protocol was not prepared. | 4 |
|  | 24c | Describe and explain any amendments to information provided at registration or in the protocol. | Not carried out |
| Support | 25 | Describe sources of financial or non-financial support for the review, and the role of the funders or sponsors in the review. | Not carried out |
| Competing interests | 26 | Declare any competing interests of review authors. | Not carried out |
| Availability of data, code and other materials | 27 | Report which of the following are publicly available and where they can be found: template data collection forms; data extracted from included studies; data used for all analyses; analytic code; any other materials used in the review. | Not carried out |

**Supplementary 2 Search strategy**

**Systematic Search Syntax for Electronic Databases (-October 2025)**

| **Pubmed** | |
| --- | --- |
| #1 | ("Inflammatory Bowel Diseases"[Mesh] OR "Crohn Disease"[Mesh] OR "Colitis, Ulcerative"[Mesh]) OR ("Inflammatory Bowel Disease"[tiab] OR "IBD"[tiab] OR "UC"[tiab] OR "Ulcerative Colitis"[tiab]) |
| #2 | ("Gastrointestinal Microbiome"[Mesh] OR "Microbiota"[Mesh]) OR ("gut microbiota"[tiab] OR "gut microbiome"[tiab] OR "intestinal microbiota"[tiab] OR "intestinal flora"[tiab] OR "gut flora"[tiab]) |
| #3 | ("Depression"[Mesh] OR "Anxiety"[Mesh] OR "Mood Disorders"[Mesh] OR "Stress, Psychological"[Mesh]) OR ("depression"[tiab] OR "depressive symptoms"[tiab] OR "anxiety"[tiab] OR "psychological stress"[tiab] OR "emotional stress"[tiab] OR "mental stress"[tiab]) |
| #4 | #1 AND #2 AND #3 |
| **Embase** | |
| #1 | ('Inflammatory Bowel Diseases'/exp OR 'Crohn Disease'/exp OR 'Ulcerative Colitis'/exp) OR ('inflammatory bowel disease'.ti,ab OR 'IBD'.ti,ab OR 'UC'.ti,ab OR 'ulcerative colitis'.ti,ab) |
| #2 | ('Gastrointestinal Microbiome'/exp OR 'Microbiota'/exp) OR ('gut microbiota'.ti,ab OR 'gut microbiome'.ti,ab OR 'intestinal microbiota'.ti,ab OR 'intestinal flora'.ti,ab OR 'gut flora'.ti,ab) |
| #3 | ('Depression'/exp OR 'Anxiety'/exp OR 'Mood Disorders'/exp OR 'Psychological Stress'/exp) OR ('depression'.ti,ab OR 'depressive symptoms'.ti,ab OR 'anxiety'.ti,ab OR 'psychological stress'.ti,ab OR 'emotional stress'.ti,ab OR 'mental stress'.ti,ab) |
| #4 | #1 AND #2 AND #3 |
| **Cochrane Library** | |
| #1 | (Inflammatory Bowel Diseases):ti,ab,kw OR (Crohn Disease):ti,ab,kw OR (Colitis, Ulcerative):ti,ab,kw OR (inflammatory bowel disease):ab OR (ulcerative colitis):ab |
| #2 | (Gastrointestinal Microbiome):ti,ab,kw OR (Microbiota):ti,ab,kw OR (gut microbiota):ab OR (gut microbiome):ab OR (intestinal flora):ab |
| #3 | (Depression):ti,ab,kw OR (Anxiety):ti,ab,kw OR (Mood Disorders):ti,ab,kw OR (Stress, Psychological):ti,ab,kw OR (psychological stress):ab |
| #4 | #1 AND #2 AND #3 |
| **Web of Science** | |
| #1 | ("Inflammatory Bowel Diseases" OR "Crohn Disease" OR "Colitis, Ulcerative" OR "inflammatory bowel disease" OR "IBD" OR "UC" OR "ulcerative colitis") |
| #2 | ("Gastrointestinal Microbiome" OR "Microbiota" OR "gut microbiota" OR "gut microbiome" OR "intestinal microbiota" OR "intestinal flora" OR "gut flora") |
| #3 | ("Depression" OR "Anxiety" OR "Mood Disorders" OR "Stress, Psychological" OR "depression" OR "depressive symptoms" OR "anxiety" OR "psychological stress" OR "emotional stress" OR "mental stress") |
| #4 | #1 AND #2 AND #3 |
| **EBSCO** | |
| #1 | ("Inflammatory Bowel Diseases"[SU] OR "Crohn Disease"[SU] OR "Colitis, Ulcerative"[SU]) OR ("Inflammatory Bowel Disease"[AB] OR "IBD"[AB] OR "UC"[AB] OR "Ulcerative Colitis"[AB]) |
| #2 | ("Gastrointestinal Microbiome"[SU] OR "Microbiota"[SU]) OR ("gut microbiota"[AB] OR "gut microbiome"[AB] OR "intestinal microbiota"[AB] OR "intestinal flora"[AB] OR "gut flora"[AB]) |
| #3 | ("Depression"[SU] OR "Anxiety"[SU] OR "Mood Disorders"[SU] OR "Stress, Psychological"[SU]) OR ("depression"[AB] OR "depressive symptoms"[AB] OR "anxiety"[AB] OR "psychological stress"[AB] OR "emotional stress"[AB] OR "mental stress"[AB]) |
| #4 | #1 AND #2 AND #3 |
| **Scopus** | |
| #1 | (Inflammatory Bowel Diseases):TITLE,ABS,KEY OR (Crohn Disease):TITLE,ABS,KEY OR (ColiTITLEs, UlceraTITLEve):TITLE,ABS,KEY OR (inflammatory bowel disease):ABS OR (ulceraTITLEve coliTITLEs):ABS |
| #2 | (GastrointesTITLEnal Microbiome):TITLE,ABS,KEY OR (Microbiota):TITLE,ABS,KEY OR (gut microbiota):ABS OR (gut microbiome):ABS OR (intesTITLEnal flora):ABS |
| #3 | (Depression):TITLE,ABS,KEY OR (Anxiety):TITLE,ABS,KEY OR (Mood Disorders):TITLE,ABS,KEY OR (Stress, Psychological):TITLE,ABS,KEY OR (psychological stress):ABS |
| #4 | #1 AND #2 AND #3 |

**Supplementary 3 Risk assessment of prospective studies using CASP checklist.**

| ID | Did the study address a clearly focused issue? | Was the cohort recruited in an acceptable way? | Was the exposure accurately measured to minimise bias? | Was the outcome accurately measured to minimise bias? | Have the authors identified all important confounding factors?Have the authors identified all | Have they taken account of the confounding factors in the design and/or analysis? | Was the follow up of subjects complete enough? | Was the follow up of subjects long enough? | What are the results of this study? | How precise are the results? | Do you believe the results? | Can the results be applied to the local population? | Do the results of this study fit with other available evidence? | What are the implications of this study for practice? | Applicability score |
| --- | --- | --- | --- | --- | --- | --- | --- | --- | --- | --- | --- | --- | --- | --- | --- |
| 1 | Yes | Yes | Can’t Tell | Yes | Can’t Tell | Yes | Can’t Tell | Can’t Tell | Yes | Yes | Yes | Can’t Tell | Yes | Yes | 9/14 |
| 2 | Yes | Yes | Can’t Tell | Yes | Can’t Tell | Yes | Can’t Tell | Can’t Tell | Yes | Yes | Yes | Can’t Tell | Yes | Yes | 9/14 |
| 3 | Yes | Yes | Can’t Tell | Yes | Can’t Tell | Yes | Can’t Tell | Can’t Tell | Yes | Yes | Yes | Can’t Tell | Yes | Yes | 9/14 |
| 4 | Yes | Yes | Can’t Tell | Yes | Can’t Tell | Yes | Can’t Tell | Can’t Tell | Yes | Yes | Yes | Can’t Tell | Yes | Yes | 9/14 |
| 5 | Yes | Yes | Can’t Tell | Yes | Can’t Tell | Yes | Can’t Tell | Can’t Tell | Yes | Yes | Yes | Can’t Tell | Yes | Yes | 9/14 |
| 6 | Yes | Yes | Can’t Tell | Yes | Can’t Tell | Yes | Can’t Tell | Can’t Tell | Yes | Yes | Yes | Can’t Tell | Yes | Yes | 9/14 |

**Supplementary 4 Quality assessment of randomized Controlled Trials (RCT) using CASP checklist**

| ID | Clear research question | Randomised allocation | Participant follow-up complete | Participant blinding | Blinding of investigators | Blinded outcome assessors | Baseline group similarity | Equal care provided | Intervention effects reported | Estimate precision reported | Benefit–risk balance | Applicability to context | Value beyond existing care | Applicability Score |
| --- | --- | --- | --- | --- | --- | --- | --- | --- | --- | --- | --- | --- | --- | --- |
| 1 | Yes | Yes | Yes | Yes | Yes | Yes | Yes | Yes | Yes | Yes | Can’t Tell | Can’t Tell | Can’t Tell | 10/13 |
| 2 | Yes | Yes | Yes | No | Yes | Yes | Yes | Yes | Yes | No | Can’t Tell | Can’t Tell | Can’t Tell | 9/13 |
| 3 | Yes | Yes | Yes | No | Yes | Yes | Yes | Yes | Yes | Yes | Can’t Tell | Can’t Tell | Can’t Tell | 9/13 |
| 4 | Yes | Yes | Yes | Yes | Yes | Yes | Yes | Yes | Yes | No | Can’t Tell | Can’t Tell | Can’t Tell | 10/13 |
